# Extra-osseous solitary hard palate neurofibroma

**DOI:** 10.1016/S1808-8694(15)31109-5

**Published:** 2015-10-19

**Authors:** Aline Cristina Batista Rodrigues Johann, Patrícia Carlos Caldeira, Giovanna Ribeiro Souto, João Batista de Freitas, Ricardo Alves Mesquita

**Affiliations:** 1 M. Sc., PhD student - Dentistry School - Federal University of Minas Gerais; 2 Undergraduate Student - Dentistry School - Federal University of Minas Gerais; 3 Undergraduate Student - Dentistry School - Federal University of Minas Gerais; 4 M. Sc. Professor - Dentistry School - Federal University of Minas Gerais; 5 PhD. Professor - Dentistry School - Federal University of Minas Gerais

**Keywords:** hard palate, mouth mucosa, neurofibroma, neurofibromatosis 1

## INTRODUCTION

The neurofibroma (NF) is a benign tumor of the peripheral nerve sheath that rarely affects the head and neck. However, among neural lesions, this is the one that most frequently affects this region^1^^,^^2^. The NF can be intra or extra-osseous, alone or multiple (associated with type I neurofibromatosis)^2^^,^^3^. The most common extra-osseous mouth NF locations are tongue, oral mucosa and lips^2^. In the literature we found two well-documented cases of solitary extra-osseous NF in the hard palate^1^^,^^4^.

## CASE PRESENTATION

**Figure 1:**
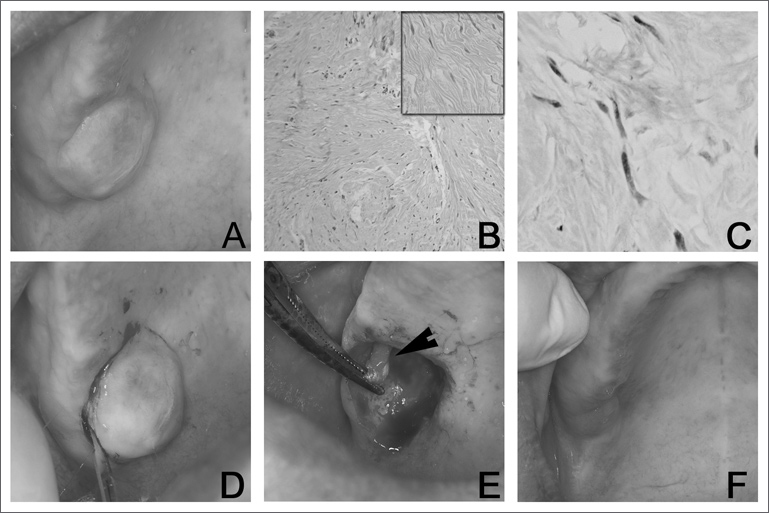
A- During clinical exam we noticed a single, asymptomatic, sessile, fibrous, pinkish, smooth nodule, measuring 30 × 30 × 05mm, on the right side of the posterior hard palate. B- Hlstopathology revealed proliferation of fusiform cells with undulated nucleus, disorganized and distributed in fibrous connective tissue (hematoxylln-eosin dye; 100X magnification). Detail image of fusiform cells with undulated nucleus [hematoxylln-eosin dye 200X magnification). C- Neoplastic cells were mmunoposltive for protein S-100 (streptoavidin-biotin technique, 400x magnification). D- During surgical excision we could see that the lesion was attached to the greater palatine nerve (arrow), and part of the nerve was also resected. F- No recurrence was seen during the 12 months of follow up.

A 39 year-old-female was referred to us because of a diagnosis of a lesion on the palate. The patient was edentulous and had a single, asymptomatic, sessile and fibrous pink and smooth nodule measuring 30 × 30 × 05mm, on the right side of the posterior region of the hard palate, near the alveolar border, that had been evolving for three years (Figure 1A). Her medical history was uneventful. X-ray images did not show any alterations. The clinical diagnosis was pleomorphic adenoma or benign mensenchymal neoplasia. We did an incisional biopsy and the specimen was referred to analysis. Histological exam showed fusiform cell proliferation with undulated nuclei distributed in a disorganized fashion on the fibrous connective tissue (Figure 1B). All the neoplastic cells were immunopositive for protein S-100 (streptoavidin-biotin technique), Dako Corporation®, clone: Z0311, dilution 1:100, without antigenic recovery, incubated for 18 hours at $4^{\underline{\text{o}}}\text{C}$) (Figure 1C). The final diagnosis was NF. The patient was re-evaluated and there were no more evidences of type I neurofibromatosis. The lesion was excised; it was well outlined and attached to the greater palatine nerve (Figure 1D). This portion of the nerve was also resected (Figure 1E). There was no recurrence during the 12 months of follow up (Figure 1F).

## DISCUSSION

Pollack^1^ and Shimoyama et al^4^ reported two cases of solitary extra-osseous hard palate NF, which usually is a small, sessile, smooth, well outlined and not-encapsulated nodule^1^^,^^2^. Cherrick and Eversole^2^ observed a predilection for females. Chen and Miller^5^ reported that mouth NF affect people between 9 and 72 years of age. These clinical characteristics were seen in this case. NFs are immunopositive for the S-100 protein in 85 to 100% of the cases, indicating its neural origin^3^^,^^6^. Treatment for solitary NF is surgical excision and recurrence is rare^2^^,^^4^. In the case hereby described the tumor was easily removed because it was well outlined. Moreover, a portion of the greater palatine nerve was also removed.

## FINAL COMMENTS

It is fundamental to follow the patient with NF, because the solitary NF can be the first manifestation of type I neurofibromatosis. This patient is under follow up care and until this report was made we did not see any relapse.
